# *In vivo* siRNA delivery of *Keap1* modulates death and survival signaling pathways and attenuates concanavalin-A-induced acute liver injury in mice

**DOI:** 10.1242/dmm.015537

**Published:** 2014-07-04

**Authors:** Águeda González-Rodríguez, Bjorn Reibert, Thomas Amann, Rainier Constien, Cristina M. Rondinone, Ángela M. Valverde

**Affiliations:** 1 Centro de Investigación Biomédica en Red de Diabetes y Enfermedades Metabólicas Asociadas (CIBERDEM), Instituto de Salud Carlos III, Spain; 2 Instituto de Investigaciones Biomédicas “Alberto Sols” (Consejo Superior de Investigaciones Científicas/Universidad Autónoma de Madrid), 28029 Madrid, Spain; 3 Roche Kulmbach GmbH, Kulmbach, D 95326, Germany; 4 Metabolic Diseases, Hoffmann-la Roche Inc., Nutley, NJ 07110-1199, USA

**Keywords:** Keap1, Nrf2, Acute liver failure, Apoptosis, *In vivo* siRNA

## Abstract

Oxidative stress contributes to the progression of acute liver failure (ALF). Transcription factor nuclear factor-erythroid 2-related factor (Nrf2) serves as an endogenous regulator by which cells combat oxidative stress. We have investigated liver damage and the balance between death and survival signaling pathways in concanavalin A (ConA)-mediated ALF using *in vivo* siRNA delivery targeting *Keap1* in hepatocytes. For that goal, mice were injected with *Keap1*- or luciferase-siRNA-containing liposomes via the tail vein. After 48 hours, ALF was induced by ConA. Liver histology, pro-inflammatory mediators, antioxidant responses, cellular death, and stress and survival signaling were assessed. *Keap1* mRNA and protein levels significantly decreased in livers of *Keap1*-siRNA-injected mice. In these animals, histological liver damage was less evident than in control mice when challenged with ConA. Likewise, markers of cellular death (FasL and caspases 8, 3 and 1) decreased at 4 and 8 hours post-injection. Nuclear Nrf2 and its target, hemoxygenase 1 (HO1), were elevated in *Keap1*-siRNA-injected mice compared with control animals, resulting in reduced oxidative stress in the liver. Similarly, mRNA levels of pro-inflammatory cytokines were reduced in livers from *Keap1*-siRNA-injected mice. At the molecular level, activation of c-jun (NH2) terminal kinase (JNK) was ameliorated, whereas the insulin-like growth factor I receptor (IGFIR) survival pathway was maintained upon ConA injection in *Keap1*-siRNA-treated mice. In conclusion, our results have revealed a potential therapeutic use of *in vivo* siRNA technology targeted to *Keap1* to combat oxidative stress by modulating Nrf2-mediated antioxidant responses and IGFIR survival signaling during the progression of ALF.

## INTRODUCTION

Acute liver failure (ALF) is a severe liver disease that frequently exhibits a fulminant progression. It is caused by alcohol consumption, drug induction [e.g. acetaminophen (APAP) overdose], viral hepatitis infection and autoimmune hepatitis. Worldwide, ALF is associated with coagulopathy, hepatic encephalopathy and kidney injury, and involves high mortality in the absence of immediate liver transplantation (Whitehouse and Wendon, 2013). Concanavalin A (ConA)-induced liver failure in mice is a widely used animal model that accurately represents ALF owing to its dependency on T-cell-mediated acute inflammation leading to hepatocyte apoptosis and necrosis (Tiegs et al., 1992).

At the molecular level, tumor necrosis factor (TNF)α and Fas/CD95 ligand (FasL) pathways are involved in the pathogenesis of the massive hepatocyte cell death during ALF (Batey and Wang, 2002; Ksontini et al., 1998; Zang et al., 2000). Moreover, inflammation-mediated oxidative stress potentiates cytokine-mediated signaling and contributes to the progression of the disease. In this regard, a tight control of reactive oxygen species (ROS) levels by antioxidant molecules is important to restore the redox balance in cells challenged with oxidative insults. Therefore, antioxidant compounds have become attractive as therapeutic agents in the treatment of ALF (Downs et al., 2012; Li and Liu, 2004; Sun et al., 2011).

The transcription factor Nrf2 (nuclear factor-erythroid 2-related factor) is a master regulator of adaptation to xenobiotic and oxidative stress and therefore is fundamental to liver physiology (Hayes et al., 2000; Kitteringham et al., 2010; Klaassen and Reisman, 2010). Nrf2 activates detoxifying enzymes by binding to antioxidant response elements (AREs) in promoters of several genes and is an endogenous regulator by which cells combat oxidative stress. Under physiological or basal conditions, Keap1-Cul3-RBX1 complex is present in the cytosol, constantly degrading Nrf2 (Dinkova-Kostova et al., 2005). Exposure to a number of endogenous or exogenous stressors leads to redox modulation of cysteines in Keap1, which dissociates Nrf2, thereby rescuing Nrf2 from proteasomal degradation and allowing for its entry into the nucleus (Kobayashi and Yamamoto, 2005). Thus, the Nrf2-regulated adaptive response represents a potential target for attenuation of inflammation by protecting against inflammatory oxidative damage and pro-inflammatory redox-sensitive signaling. Of relevance, Nrf2-deficient mice are highly susceptible to liver injury induced by various hepatotoxic agents, including ConA and APAP (Enomoto et al., 2001; Liu et al., 2013; Osburn et al., 2008).

On the other hand, ALF also triggers survival pathways to counteract death signaling. Activation of hepatocyte growth factor (HGF) – which can be transiently produced by T cells upon CoA stimulation (Iwai et al., 1992) – the phosphatidylinositol 3 kinase (PI3K)-BclxL axis or nuclear factor kappa B (NFκB) signaling pathways can ameliorate liver injury (Luedde et al., 2006; Mobasher et al., 2013; Moriya et al., 2012; Zhu et al., 2012). Moreover, insulin-like growth factor I receptor (IGFIR) triggers survival responses through IGFIR-IRS1/2-Akt-mediated signaling in hepatocytes (Tovar et al., 2010). An imbalance between inflammatory and/or stress and protective pathways might trigger in hepatic failure, so these signals should be very tightly regulated to maintain hepatic homeostasis.

**TRANSLATIONAL IMPACT**

**Clinical issue**

Acute liver failure (ALF) is a severe liver disease that frequently exhibits a fulminant progression and involves high mortality in the absence of immediate liver transplantation. Previous elegant studies in transgenic mice reported the involvement of the transcription factor Nrf2 in ALF progression. The activity of this factor is key for the regulation of oxidative stress and for liver physiology; thus, its pathway might be potentially targeted as a pharmacological approach against ALF.

**Results**

In this study, the authors designed a specific short interfering RNA (siRNA) against the mRNA of *Keap1* (an Nrf2 suppressor) for further *in vivo* administration. Mice were injected with *Keap1*- or luciferase (control)-siRNA-containing liposomes via the tail vein and, after 48 hours, ALF was induced by using concanavalin A (ConA), a known hepatotoxic agent. Silencing of *Keap1* attenuated ConA-induced inflammatory-associated liver damage; this effect was due to the decrease of oxidative stress by the enhancement of the Nrf2-mediated antioxidant response and the maintenance of IGFIR (insulin-like growth factor I receptor) survival signaling during the progression of ALF induced by ConA.

**Implications and future directions**

RNA interference using siRNA has become the next frontier in molecular medicine and promises larger advantages compared with other drug-development strategies owing to its easier design, higher target selectivity and lower toxicity. This study revealed a potential therapeutic use in ALF of *in vivo* siRNA technology targeted to oxidative stress regulators.

RNA interference using short interfering RNA (siRNA) has become not only an exciting new tool in molecular biology but also the next frontier in the molecular medicine and promises larger advantages to other drug-development strategies owing to its easier design, higher target selectivity and lower toxicity (Kawakami and Hashida, 2007; Ryther et al., 2005). Regarding application in hepatotoxicity, previous studies have demonstrated that siRNA therapy is effective in reducing the expression of caspase 8 (Zender et al., 2003) or Fas (Jiang et al., 2012; Song et al., 2003) in mouse models of ALF.

On that basis, in this study we have analyzed the balance between death and survival signaling pathways involved in ConA-mediated ALF using an *in vivo* siRNA delivery system that specially targets *Keap1* in hepatocytes.

## RESULTS

### Reduction of *Keap1* in the liver by *in vivo* siRNA administration reduces ConA-mediated apoptosis

Because oxidative stress plays a crucial role in the progression of ALF, we have investigated whether promoting Nrf2 antioxidant signaling would be an important intervention to protect liver against ConA-induced ALF. Keap1 is the main Nrf2 suppressor, so we designed a specific siRNA against *Keap1* mRNA for further *in vivo* administration.

**Fig. 1.**
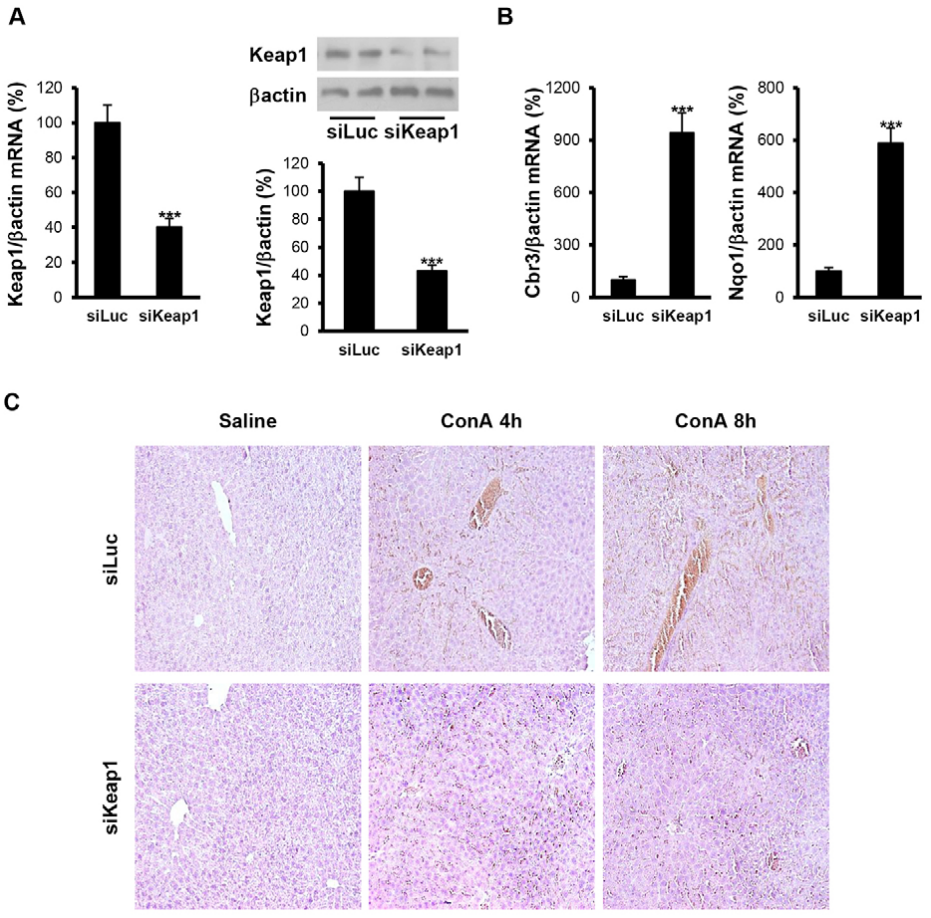
**Reduction of *Keap1* in the liver by siRNA administration *in vivo* attenuates ConA-induced liver damage.** Analysis of *Keap1* expression and histopathological features in liver samples from luciferase siRNA (siLuc) or *Keap1* siRNA (siKeap1) mice after 4 and 8 hours of ConA treatment (*n*=4–6 animals per condition). (A) (left panel) *Keap1* mRNA levels determined by real-time PCR. (Right panel) Representative blots with the indicated antibodies and quantification of the densitometric analysis from all blots. Data are presented as mean±s.e.m. relative to siLuc mice. (B) *Cbr3* and *Nqo1* mRNA levels determined by real-time PCR. Data are presented as mean±s.e.m. relative to siLuc mice. (C) Representative images from hematoxylin and eosin staining. ***P<0.005, siKeap1 versus siLuc.

Firstly, we verified that *Keap1* siRNA treatment was capable of downregulating *Keap1* gene and protein expression in the liver. For this goal, we injected control (luciferase)- or *Keap1*-siRNA-containing liposomes into the tail vein of mice and, after 48 hours, livers were harvested for mRNA and protein isolation. As Fig. 1A shows, both *Keap1* mRNA and protein expression were reduced by 65% in the livers of *Keap1*-siRNA-injected mice compared with control mice (receiving the luciferase siRNA). Moreover, we analyzed the expression of two Nrf2-dependent genes and, accordingly with Keap1 downregulation, *Cbr3* (NADPH carbonyl reductase 3) and *Nqo1* (NADPH quinone oxidoreductase 1) mRNA expression increased in the livers from *Keap1*-siRNA-injected mice as compared with mice injected with luciferase siRNA (Fig. 1B).

Once we had verified the efficacy of this strategy to reduce Keap1 levels in the liver after 48 hours of siRNA application, we injected the mice with ConA [25 mg/kg body weight, intravenous (i.v.) tail vein injection] and examined the histopathology of the livers. After 4 hours, luciferase-siRNA-injected mice had inflammatory cell infiltrates surrounding the portal and central veins, and extensive liver damage, parenchymal necrosis and hemorrhage were evident at 8 hours post ConA injection. By contrast, livers from most *Keap1*-siRNA-injected mice demonstrated significant attenuation of all of these pathological changes associated with the hepatotoxic process (Fig. 1C). Of note, no significant differences between experimental groups in liver to body mass ratio were found after ConA injection (results not shown).

**Fig. 2.**
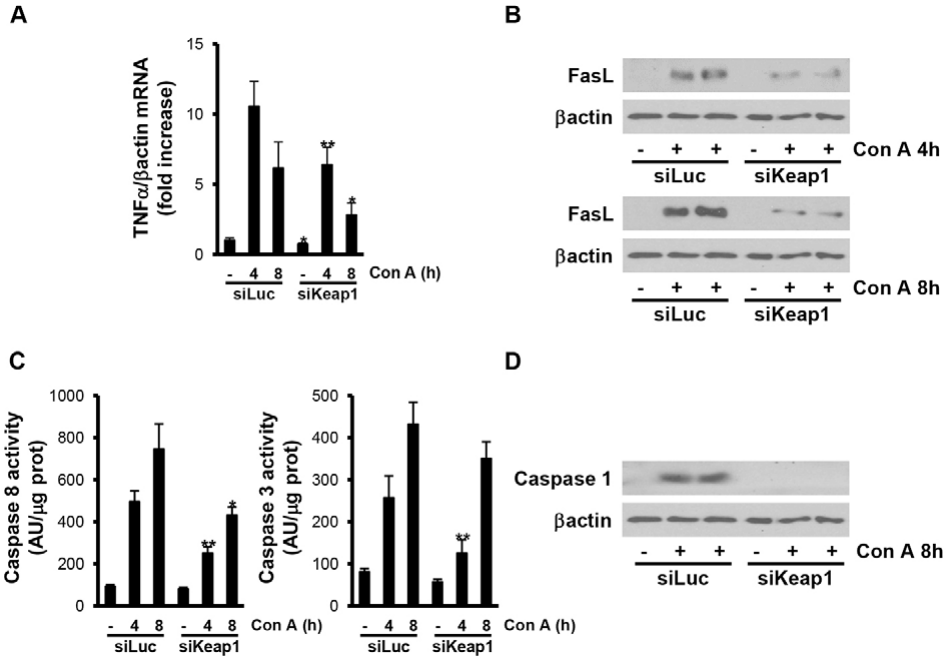
**Silencing of *Keap1* in the liver by siRNA administration *in vivo* reduces ConA-mediated apoptosis.** Analysis of cell-death markers in liver samples from luciferase siRNA (siLuc) or *Keap1* siRNA (siKeap1) mice after 4 and 8 hours of ConA treatment (*n*=4–6 animals per condition). (A) *TNFα* mRNA levels determined by real-time PCR. Data are presented as mean±s.e.m. relative to siLuc mice. (B) Representative blots with the indicated antibodies. (C) Graphics of caspase 3 and 8 enzymatic activities. Data are presented as mean±s.e.m. relative to siLuc mice. (D) Representative blots with the indicated antibodies. *P<0.05 and **P<0.01, siKeap1 versus siLuc.

It is well established that TNFα and FasL, both triggers of the death-receptor-mediated apoptotic pathways, play an important role in ConA-induced apoptosis and necrosis. Therefore, we analyzed the expression of these two ligands after ConA challenge in our mouse models. Silencing of *Keap1* significantly reduced the increase of *TNFα* mRNA levels and FasL protein expression (Fig. 2A,B). Consequently, the activation of caspase 8 and caspase 3 after ConA treatment (4 and 8 hours) was significantly ameliorated (Fig. 2C). Furthermore, the active fragment of caspase 1, a downstream mediator in Fas apoptotic signaling in response to excessive inflammation (Su et al., 2013), was only detected in livers from luciferase-siRNA-injected mice at 8 hours post-injection (Fig. 2D).

### ConA-induced oxidative stress is reduced in livers from mice injected with *Keap1* siRNA, through the enhancement of Nrf2 nuclear accumulation

**Fig. 3.**
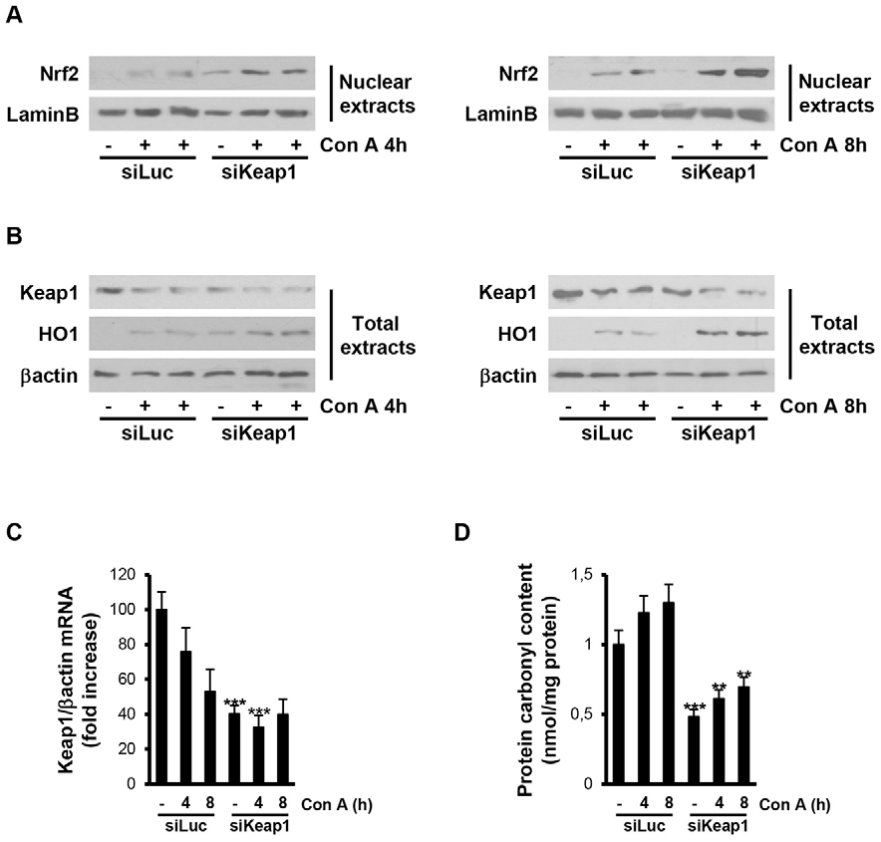
**ConA-induced oxidative stress is reduced in livers from mice injected with *Keap1* siRNA.** Analysis of Nrf2 signaling and oxidative stress markers in liver samples from luciferase siRNA (siLuc) or *Keap1* siRNA (siKeap1) mice after 4 and 8 hours of ConA treatment (*n*=4–6 animals per condition). (A,B) Representative blots with the indicated antibodies. (C) *Keap1* mRNA levels determined by real-time PCR. Data are presented as mean±s.e.m. relative to siLuc mice. (D) Graphic of carbonylated protein levels. Data are presented as mean±s.e.m. relative to siLuc mice. **P<0.01 and ***P<0.005, siKeap1 versus siLuc.

Next, we examined differences in nuclear levels of Nrf2 in mice injected with luciferase or *Keap1* siRNAs. As shown in Fig. 3A, nuclear Nrf2 was absent in livers of mice injected with luciferase siRNA before injection, but were detected at 4 and 8 hours after ConA treatment. In livers from mice injected with *Keap1* siRNA, basal nuclear Nrf2 was detected before ConA treatment and further increased at 4 and 8 hours after injection. Notably, in these mice, the total levels of Nrf2 in the nuclear compartment after ConA injection were higher than those of mice injected with luciferase siRNA. In agreement with these results, *Keap1* mRNA and protein levels were decreased in livers from mice injected with luciferase siRNA after 4 and 8 hours of ConA treatment and further decreased in mice treated with *Keap1* siRNA, reaching lower levels than in luciferase siRNA mice (Figs 3B,C). Then, we analyzed heme oxygenase 1 (HO1) expression as an Nrf2 target gene and, as depicted in Fig. 3B, ConA-induced HO1 protein content was higher in mice injected with *Keap1* siRNA compared with controls. To reinforce these data, protein carbonyl levels were measured as a broad indicator of tissue oxidative stress. ConA treatment increased protein carbonyl levels in livers from control mice injected with luciferase siRNA, whereas basal levels of carbonylated proteins were lower in livers from the *Keap1* siRNA group and were less elevated after ConA injection compared with the luciferase siRNA mice (Fig. 3D).

### Effect of *Keap1* silencing on the activation of stress- and survival-mediated signaling pathways in the livers of ConA-injected mice

**Fig. 4.**
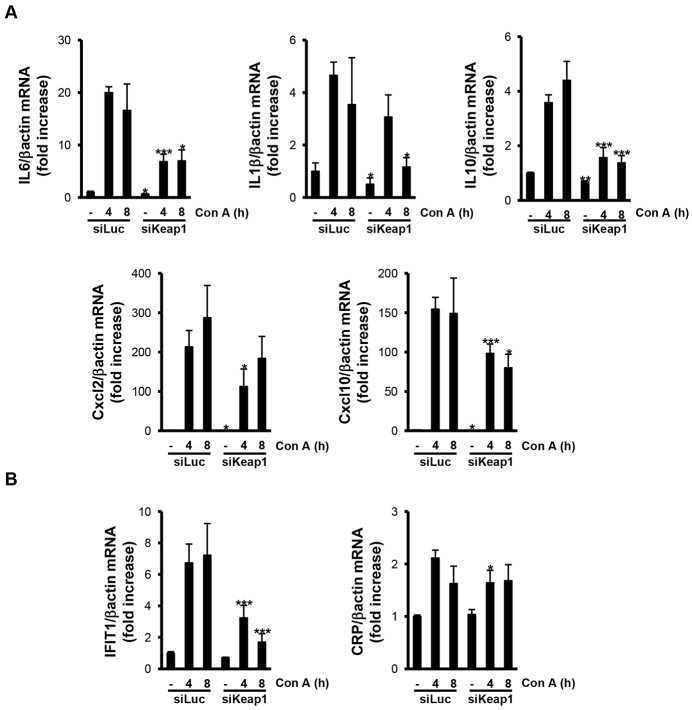
**Silencing of *Keap1* reduced the expression of inflammatory markers in ConA-injected livers.** Analysis of inflammatory markers in liver samples from luciferase siRNA (siLuc) or *Keap1* siRNA (siKeap1) mice after 4 and 8 hours of ConA treatment (*n*=4–6 animals per condition). (A,B) mRNA levels of the indicated cytokines and pro-inflammatory markers determined by real-time PCR. Data are presented as mean±s.e.m. relative to siLuc mice. *P<0.05, **P<0.01 and ***P<0.005, siKeap1 versus siLuc.

In addition to TNFα and FasL, other cytokines and chemokines are also secreted by T cells after ConA challenge and are involved in the development of ALF. In this regard, the increases of *IL6*, *IL1β*, *IL10*, *Cxcl2* and *Cxcl10* mRNA levels were significantly higher in livers from mice injected with luciferase siRNA than in livers from those mice injected with *Keap1* siRNA at 4 and 8 hours post ConA injection. Moreover, other inflammatory markers such as *IFIT1* (interferon-induced protein with tetratricopeptide repeats 1) and *CRP* (C reactive protein) followed the same pattern of response to ALF (Fig. 4A,B).

**Fig. 5.**
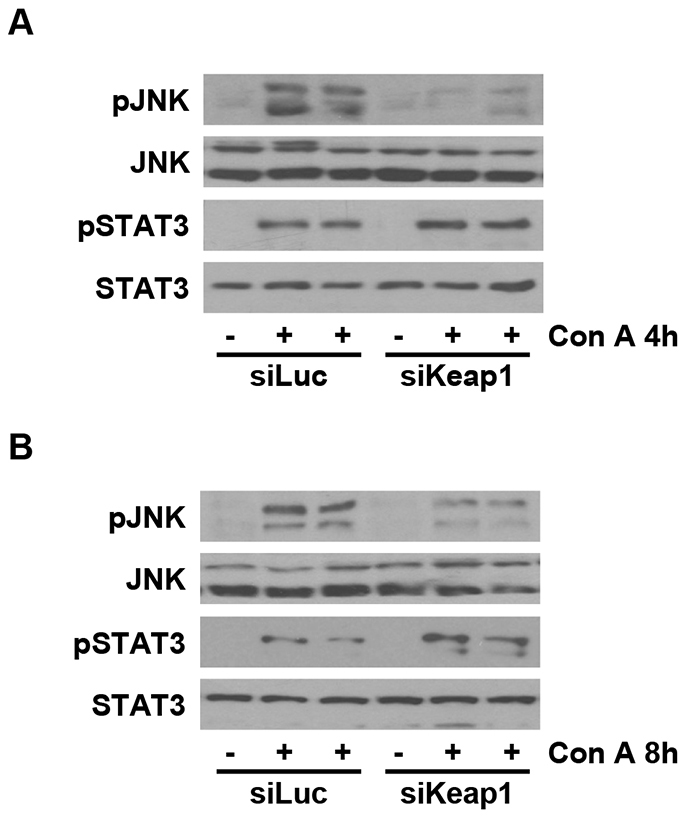
**Reduction of *Keap1* decreased the activation of JNK in ConA-injected livers.** Analysis of stress kinase activation in liver samples from luciferase siRNA (siLuc) or *Keap1* siRNA (siKeap1) mice after 4 and 8 hours of ConA treatment (*n*=4–6 animals per condition). (A,B) Representative blots with the indicated antibodies.

Next, we analyzed stress-mediated signaling in the livers from the two experimental groups in response to ConA. Activation of c-jun (NH2) terminal kinase (JNK) was detected at 4 and 8 hours post-injection in livers from ConA-treated mice injected with luciferase siRNA, whereas silencing of *Keap1* attenuated this effect (Fig. 5A,B). Moreover, signal transducer and activator of transcription 3 (STAT3) phosphorylation was detected in livers from both groups of mice at 4 and 8 hours post-injection. Interestingly, STAT3 phosphorylation was significantly higher in livers from *Keap1*-siRNA-injected mice.

**Fig. 6.**
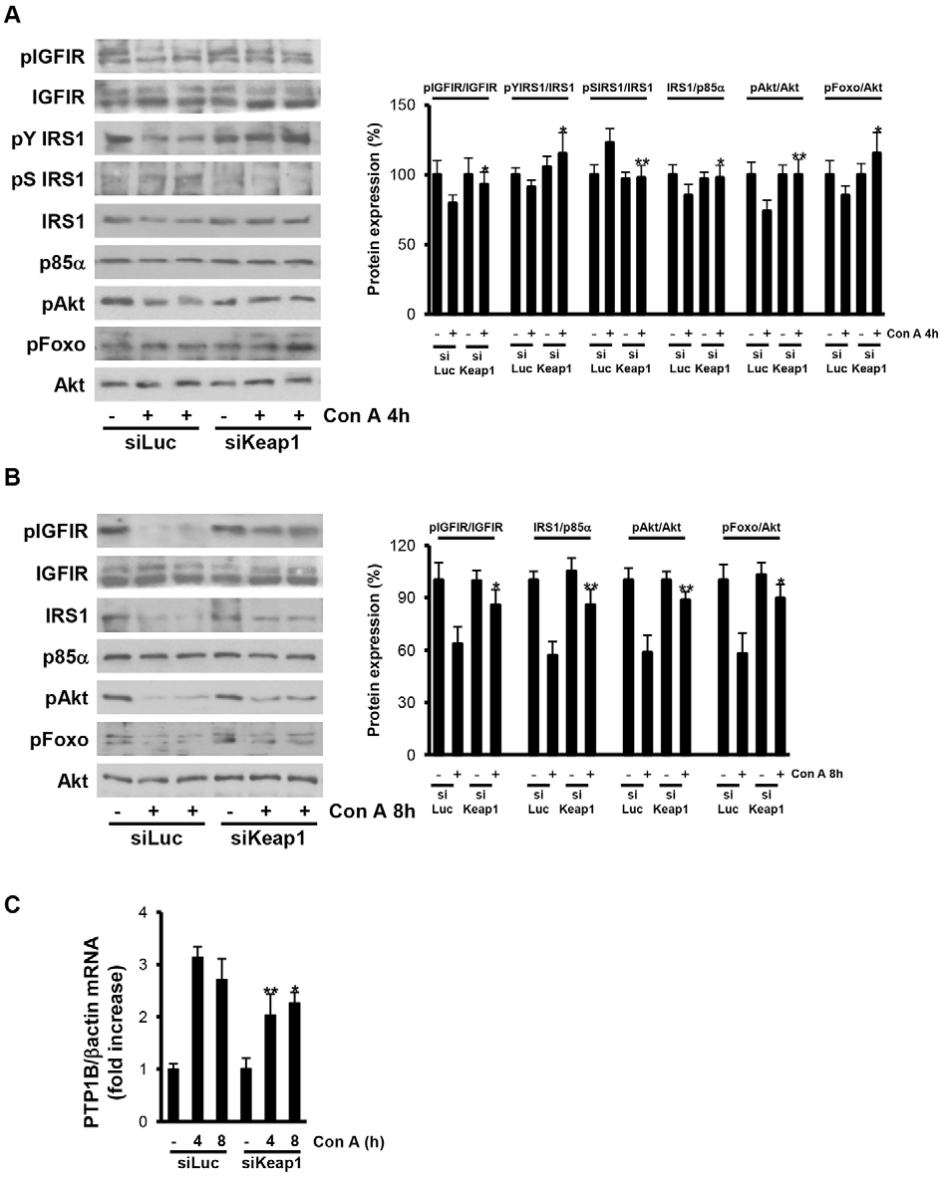
**Reduction of *Keap1* preserved the IGFIR survival signaling pathway in ConA-injected livers.** Analysis of IGFIR survival signaling in liver samples from luciferase siRNA (siLuc) or *Keap1* siRNA (siKeap1) mice after 4 and 8 hours of ConA treatment (*n*=4–6 animals per condition). (A,B) Representative blots with the indicated antibodies and quantification of the densitometric analysis from all blots. Data are presented as mean±s.e.m. relative to siLuc mice. (C) *PTP1B* mRNA levels determined by real-time PCR. Data are presented as mean±s.e.m. relative to siLuc mice. *P<0.05 and **P<0.01, siKeap1 versus siLuc.

Regarding survival signaling, we monitored the IGFIR pathway because of its effect in triggering survival responses in hepatocytes (Tovar et al., 2010). IGFIR tyrosine phosphorylation, IRS1 tyrosine phosphorylation and total IRS1 protein levels were barely reduced in livers from mice injected with luciferase siRNA, after 4 hours of ConA treatment, but were preserved in livers from *Keap1* siRNA animals. Likewise, the phosphorylation of Akt and its downstream substrate Foxo1 were slightly decreased in livers from luciferase-siRNA-injected mice after 4 hours of ConA treatment, but maintained in livers from *Keap1*-siRNA-injected animals (Fig. 6A). Because it is well established that activated JNK phosphorylates IRS1 at serine 307 and targets IRS1 for proteasomal degradation (Aguirre et al., 2000), we analyzed this phosphorylation and it was only detected in ConA-treated luciferase siRNA mice at 4 hours post-injection, and not in *Keap1* siRNA mice. Importantly, after 8 hours of ConA treatment, IGFIR, Akt and Foxo1 phosphorylations were completely blunted, in parallel to the degradation of IRS1 in livers from luciferase-siRNA-injected mice, but these effects were significantly attenuated in livers from the *Keap1* siRNA group (Fig. 6B).

Finally, we analyzed the expression of PTP1B as a negative modulator of IGFIR-mediated survival signaling (Buckley et al., 2002). After ConA injection, *PTP1B* mRNA levels were augmented in livers from mice injected with luciferase siRNA; however, ConA-induced PTP1B content was reduced in livers from *Keap1*-siRNA-injected animals (Fig. 6C).

## DISCUSSION

The identification of currently unknown protective mechanisms against immune-mediated hepatocyte death might help in the development of new approaches to block the progression of ALF. Various studies have demonstrated that ligands of death receptors, inflammation-mediated oxidative stress and cytokines play a crucial role in the progression of ConA-induced ALF (Ksontini et al., 1998; Sun et al., 2011; Tagawa et al., 1998). However, the extent of the imbalance between stress and survival signaling pathways triggered during ConA-induced liver damage remains largely unknown.

Previously, it was shown that Nrf2 deficiency in mice conferred susceptibility to ConA-induced liver failure and, on the other hand, amplification of Nrf2 effects by conditional deletion of Keap1 in hepatocytes (c*Keap1* KO) protected against ConA-mediated inflammatory liver injury (Osburn et al., 2008). More recently, Ke and co-workers (Ke et al., 2013) found a cytoprotective mechanism by which *Keap1* deficiency in hepatocytes prevented oxidative damage in a model of ischemia/reperfusion injury followed by orthotopic liver transplantation. Besides these elegant studies in transgenic mice, our present results have unraveled that manipulation of Nrf2 by means of administration of liposomes bearing siRNA oligonucleotides directed to *Keap1* in the liver attenuates ConA-induced inflammatory-associated liver damage. This technique efficiently allowed the release of Nrf2 for its nuclear translocation: higher levels of nuclear Nrf2 were detected under basal conditions and after 4 and 8 hours of ConA treatment in the livers of mice injected with *Keap1* siRNA compared with control mice (injected with luciferase siRNA). Moreover, we also monitored the activation of Nrf2 by analyzing the expression of ARE-dependent antioxidant genes such as *Cbr3* and *Nqo1*, and HO1 protein levels, which were increased in the livers from *Keap1*-siRNA-injected mice compared with the control group. As a broad indicator of tissue oxidative stress, protein carbonyl levels were lower in livers from the *Keap1*-siRNA-injected group at both basal conditions and after ConA treatment.

ConA-induced apoptosis was attenuated in *Keap1*-siRNA-injected mice in parallel with a decrease in both FasL expression and caspase 8 activation, which constitute two of the crucial nodes of the death-receptor (extrinsic) apoptotic pathway involved in the effects of ConA (Seino et al., 1997). In the light of these results, several studies have demonstrated that delivery of siRNA oligonucleotides targeting Fas or caspase 8 successfully reduced liver damage (Jiang et al., 2012; Song et al., 2003; Zender et al., 2003), reinforcing our data on the benefit of silencing *Keap1*. A step further, *Keap1* siRNA also decreased the elevation of the caspase 1 active fragment (p10) upon ConA challenge, suggesting that the inflammasome is also a target of the Keap1-Nrf2 axis. In fact, a recent report has shown protection against D-galactosamine- and lipopolysaccharide-induced acute inflammation and liver failure by HO1 overexpression through suppression of the NLRP3 signaling pathway (Kim and Lee, 2013).

Previous studies have demonstrated that the pathogenesis of ConA-induced hepatitis is mainly mediated by the release of inflammatory cytokines (Ksontini et al., 1998; Tiegs et al., 1992). Lower levels of cytokines and other inflammatory markers were found in livers from *Keap1*-siRNA-injected mice after ConA treatment compared with the control group. At the molecular level, we have shown for the first time the modulation of intracellular signaling pathways in the liver through decreasing *Keap1* levels, which was not investigated in the transgenic animal models. In this regard, reduced stress and/or proinflammatory signaling triggered by JNK activation was evident in *Keap1*-siRNA-injected mice upon ConA challenge. Because JNK activation depends in part on both high cytokine expression and ROS levels (Seki et al., 2012), a decrease in JNK phosphorylation agrees with reduced oxidative stress. By contrast, STAT3 phosphorylation was increased after ConA injection in livers from *Keap1*-siRNA-injected mice, although *IL6* levels were lower in these mice compared with the control luciferase-siRNA-injected mice. It is well known that the IL6-STAT3 pathway participates in anti-inflammatory, survival and regenerative (after partial hepatectomy) signaling pathways (Cressman et al., 1995; Subramaniam et al., 2013; Wegenka et al., 1994). However, besides IL6, STAT3 can be activated through other pathways, such as that of IGFIR signaling. In particular, some studies have demonstrated that IGFIR can directly phosphorylate the Janus kinases (JAK) 1 and 2, leading to the phosphorylation and subsequent activation of STAT3 (Himpe and Kooijman, 2009; Zong et al., 2000). Because the phosphorylation of IGFIR was maintained in livers from *Keap1* siRNA mice, but strongly decreased in livers from luciferase-siRNA-injected mice, this event might explain the differences in the levels of STAT3 phosphorylation found between the two groups of animals. Moreover, recent data from our laboratory demonstrated that IGFIR triggers survival responses in hepatocytes in a model of APAP-induced ALF (Mobasher et al., 2013). The results presented herein clearly show that a reduction of *Keap1* prevented the drop of the IGFIR-IRS1-Akt-Foxo-mediated survival pathway in ConA-treated mice. In fact, this is the first study demonstrating that the IGFIR survival pathway gradually decreases during ConA-induced ALF. Mechanistically, this might be due to the combination of: (1) the JNK-mediated feedback mechanism on IGFIR signaling leading to IRS1 serine 307 phosphorylation that precedes its degradation (Aguirre et al., 2000) with (2) the upregulation of PTP1B by the elevation of proinflammatory cytokines resulting in dephosphorylation and inactivation of IGFIR (Buckley et al., 2002). The latter effect was also reduced in the livers from *Keap1*-siRNA-injected mice compared with luciferase siRNA animals after ConA-treatment, suggesting a newly identified role for PTP1B in ALF induced by ConA.

In conclusion, our results have revealed a potential therapeutic use of *in vivo* siRNA technology targeted to *Keap1* to decrease oxidative stress by the enhancement of the Nrf2-mediated antioxidant response and the maintenance of the IGFIR survival signaling during the progression of ALF induced by ConA.

## MATERIALS AND METHODS

### Reagents

TRIzol reagent and SuperScript™ III First-Strand Synthesis System were obtained from Invitrogen (Gran Island, NY). Bovine serum albumin and ConA were from Sigma-Aldrich (St Louis, MO). Bradford reagent, acrylamide and Immunoblot PVDF membrane were from Bio-Rad (Madrid, Spain). Immobilon Western Chemiluminescent HRP Substrate was purchased from Millipore (Billerica, MA).

### Antibodies

The antibodies used were: anti-phospho JNK (Thr183/Tyr185) (#4668), anti-phospho STAT3 (Tyr705) (#9131), anti-STAT3 (#8719), anti-phospho p38 MAPK (Thr180/Tyr182) (#9211), anti-p38 MAPK (#9212), anti-phospho Foxo1 (#9461) and anti-Akt (#9272) from Cell Signaling Technology (MA, USA); anti-phospho IGFIR (Tyr1165/1166) (sc-101704), anti-JNK (sc-571), anti-phospho-Akt1/2/3 (Ser473) (sc-7985-R), anti-caspase 1 (sc-514), anti-Nrf2 (sc-722) and anti-Keap1 (sc-33569) from Santa Cruz (Palo Alto, CA); anti-phospho IRS1 (Tyr1179) (07-844), anti-phospho IRS1 (Ser 307) (07-247), anti-IRS1 (06-248), anti-p85α (06-195) and anti-HO1 (AB1284) antibodies from Merck Millipore (Merck KGaA, Darmstadt, Germany); anti-β-actin (A-5441) antibody from Sigma Chemical Co. (St Louis, MO); anti-Lamin B (aB16048) and FasL (aB68338) from Abcam (Abcam, Cambridge, UK). Anti-IGFIR antibody was a gift of S. Pons (CSIC, Spain).

### Animals

8-week-old male C57BL/6 mice purchased from Charles River Laboratories (Charles River, Barcelona, Spain) were maintained in light/dark (12-hour light/12-hour dark)-, temperature (22°C)- and humidity-controlled rooms, and fed *ad libitum* with free access to drinking water. All animal experimentation was controlled following the recommendations of the Federation of European Laboratory Animal Science Associations (FELASA) on health monitoring, whereas use of animals in experimental procedures was approved by the Ethical Committee at Consejo Superior de Investigaciones Científicas (CSIC) Animal Care and Use Committee.

### Acute liver damage prevention model

Mice were injected with control siRNA (luciferase siRNA, siLuc) or *Keap1* siRNA (siKeap1) (2 mg/kg body weight) via the tail vein. siRNA oligonucleotides were synthesized by Kulmbach for gene silencing of mouse *Keap1* and conjugated with a liposomal formulation of SNALP (stable nucleic acid lipid particles) previously described in detail (Zimmermann et al., 2006). After 48 hours, mice were injected with ConA (25 mg/kg body weight) via the tail vein and sacrificed 4 or 8 hours post-injection. Livers and blood samples were collected at these time periods. siRNA sequences are: Keap1 (sense) AUAUCUACAUGCACUUCGGdTsdT; Keap1 (antisense) CCGAAGUGCAUCUAGAUAUdTsdT; luciferase (sense) CUUACGCUGAGUACUUCGAdTsdT; luciferase (antisense) UCGAAGUACUCAGCGUAACdTsdT.

### Liver histology

Paraffin-embedded liver biopsy sections (5 μm) were stained with hematoxylin and eosin and evaluated by a single-blinded hepatopathologist.

### Determination of protein carbonyl content

Protein oxidation of liver homogenates was measured as carbonyl group content according to the method of Richert et al. (Richert et al., 2002).

### Quantitative real-time PCR analysis and primer sequence

Total RNA was extracted with Trizol (Invitrogen) and reverse transcribed using a SuperScript™ III First-Strand Synthesis System for qPCR following the manufacturer’s indications (Invitrogen). qPCR was performed with an ABI 7900 sequence detector using the SyBr Green method and d(N)_6_ random hexamer. Primer sequences are available upon request.

### Analysis of caspase 3 and caspase 8 activities

Caspase 3 and 8 activities were determined using caspase 3 or caspase 8 fluorescent assay kit (Clontech), respectively, following the manufacturer’s instructions as previously described (González-Rodriguez et al., 2009).

### Preparation of total protein liver extracts

Liver biopsy samples were homogenized in 10 volumes (w/v) of cold lysis buffer (50 mM Tris-HCl, 1% Triton X-100, 2 mM EGTA, 10 mM EDTA acid, 100 mM NaF, 1 mM Na_4_P_2_O_7_, 2 mM Na_3_VO_4_, 100 μg/ml phenylmethylsulphonyl fluoride, 1 μg/ml aprotinin, 1 μg/ml pepstatin A and 1 μg/ml leupeptin) using a Brinkman PT 10/35 Polytron (American Laboratory Trading Inc., East Lyme, CT). Liver extracts were cleared by microcentrifugation at 40,000 ***g*** for 30 minutes at 4°C. The supernatants were aliquoted and stored at −70°C.

### Extraction of nuclear and cytosolic protein liver extracts

Liver biopsy samples were homogenized in 10 volumes (w/v) of cold buffer A (10 mM Hepes-KOH, pH 7.9, 1.5 mM MgCl_2_, 10 mM KCl, 0.5 mM DTT, 0.2 mM PMSF, 0.75 μg/ml leupeptin, 0.75 μg/ml aprotinin) using a Brinkman PT 10/35 Polytron, allowed to swell on ice for 10 minutes and vortexed for 10 seconds. Samples were centrifuged and the supernatant containing the cytosolic fraction was stored at −70°C. The pellet containing the nuclear fraction was resuspended in cold buffer C (20 mM Hepes-KOH, pH 7.9, 25% glycerol, 420 mM NaCl, 1.5 mM MgCl_2_, 0.2 mM EDTA, 0.5 mM DTT, 0.2 mM PMSF, 0.75 μg/ml leupeptin, 0.75 μg/ml aprotinin) and incubated on ice for 20 minutes for high salt extraction. Cellular debris was removed by centrifugation for 2 minutes at 4°C and the supernatant fraction was stored at −70°C.

### Western blot analysis

After protein content determination with Bradford reagent (Bio-Rad), total protein samples were boiled in Laemmli sample buffer and submitted to 8–15% SDS-PAGE. After SDS-PAGE, gels were transferred to Immobilon membranes and were blocked using 5% non-fat dried milk or 3% BSA in 10 mM Tris-HCl, 150 mM NaCl (pH 7.5) and incubated overnight with the indicated antibodies in 0.05% Tween-20, 10 mM Tris-HCl and 150 mM NaCl (pH 7.5). Immunoreactive bands were visualized using the ECL western blotting protocol (Millipore). Densitometric analysis of the bands was performed using ImageJ software.

### Data analysis

Data are presented as mean ± s.e.m., and were compared by using Bonferroni ANOVA test. All statistical analyzes were performed using the IBM SPSS Statistics 21.0 (SPSS Inc. IBM, Armonk, NY) software with 2-sided tests, with a *P*-value of <0.05 considered as statistically significant.
